# Beyond Looking for the Rewarded Target: The Effects of Reward on Attention in Search Tasks

**DOI:** 10.3389/fpsyg.2021.632442

**Published:** 2021-02-17

**Authors:** Annabelle Walle, Michel D. Druey

**Affiliations:** Department of Psychology, University of Konstanz, Konstanz, Germany

**Keywords:** monetary reward, visual search, task demand, attentional effort, associative learning, attention

## Abstract

One puzzling result in training-test paradigms is that effects of reward-associated stimuli on attention are often seen in test but not in training. We focus on one study, where reward-related performance benefits occur in the training and which was discussed contentiously. By using a similar design, we conceptually replicated the results. Moreover, we investigated the underlying mechanisms and processes resulting in these reward-related performance benefits. In two experiments, using search tasks and having participants perform the tasks either with or without individually adjusted time pressure, we disentangled the mechanisms and processes contributing to the reward-related benefits. We found evidence that not only search efficiency is increased with increasing reward, but also that non-search factors contribute to the results. By also investigating response time distributions, we were able to show that reward-related performance effects increased as search time increased in demanding tasks but not in less demanding tasks. Theoretical implications of the results regarding how reward influences attentional processing are discussed.

## Introduction

No matter what we do, be it grocery shopping, writing an exam, or driving a car, we have to focus on the information that is currently important. Crucial for finding and selecting relevant and discarding irrelevant information is attention (e.g., Desimone and Duncan, 1995). Attention has long been considered as a multi-faceted construct resulting from the interplay of top-down, goal-directed (e.g., Folk et al., 1992; Found and Müller, 1996), and bottom-up, stimulus-driven (e.g., Theeuwes, 1992; Itti and Koch, 2000) information processing and selection. Recently, selection history has been added as an important component (Awh et al., 2012). It comprises attentional biases reflecting past selection experiences, where a subcategory deals with the attention-grabbing effect of stimuli that have been associated with reward (cf. Awh et al., 2012; Failing and Theeuwes, 2018).

### Reward-Driven Attentional Capture in Training-Test Paradigms

The effects of reward associations on attention are usually investigated in training-test paradigms (e.g., Anderson, 2013; Anderson et al., 2013; Marchner and Preuschhof, 2018), in which reward associations are first established in a training phase. The effects of these associations are then examined in a subsequent test phase.

In the training phase, search tasks are often used, where differently colored circles serve as stimuli, each containing a line of different orientation. A popular implementation of this task (e.g., Anderson et al., 2011a) requires participants to search for a target circle of a pre-specified color and to decide whether the line inside that circle is oriented horizontally or vertically. Crucially, each color of the target circle is associated with a certain reward.

To assess to what extent the associated rewards affect attention, a different search task (called *additional-singleton paradigm*; Theeuwes, 1991, 1992) has often been used in the test phase. In this task, participants search for a shape singleton among distractors (e.g., a diamond among circles) and indicate the orientation of the line inside this singleton (e.g., Anderson et al., 2011a). In some trials, however, one of the distractors appears in a color that has been associated with a certain reward in the training phase. Although reward is no longer provided, responding to the singleton target is slowed in the presence of a reward-associated distractor, and the slowing is often proportional to the associated reward (e.g., Anderson et al., 2011a; Roper et al., 2014; but see Sha and Jiang, 2016, for contrary results). This effect is called *value-driven attentional capture* (VDAC; Anderson et al., 2011a).

The reward effects in the test phase indicate that the color-reward associations have successfully been established during the training phase. Surprisingly, though, in the majority of experiments, no reward effects have been observed during this phase (e.g., Anderson et al., 2011b, 2012, 2013; Anderson, 2015a,b; Gong et al., 2016, Experiment 2; Anderson and Halpern, 2017; Roper et al., 2014; Kim and Beck, 2020). Only in a few studies, some effects of the reward-association learning on performance have been reported (e.g., Anderson and Yantis, 2012; Anderson et al., 2016; Sha and Jiang, 2016).

But how can the absence of reward effects in the training phase of many studies be explained? The study of Sha and Jiang (2016) may provide some hints for answering this question. Whereas in most VDAC studies, the line inside the target varied within a different set of orientations (horizontal/vertical) than the lines inside the distractors (tilted to the left or right, e.g., Roper et al., 2014), in the study of Sha and Jiang (2016), the line orientation inside the target and the distractors varied within the same set (horizontal/vertical). By using the same set of orientations, reward effects were also found in the training phase. Responses were faster to targets associated with high reward than to those associated with no or low reward. Interestingly, Sha and Jiang (2016) found no reward effects in the test phase. However, they found that searching for a target color which was not associated with reward in the learning phase also resulted in attentional capture by a stimulus in this color if presented as a distractor in the test phase. Based on these results, Sha and Jiang (2016) questioned the role of previously learned reward associations for attention. This criticism, though, was challenged by Anderson and Halpern (2017), who argued that the results of Sha and Jiang (2016) might either mirror a Type-II error or be due to deviations in their experimental procedure compared to most of the other studies.

Although Anderson and Halpern (2017) mainly referred to the results from the test phase, it is important to note that Sha and Jiang’s (2016) results already differed in the training phase in that they observed reliable reward-related capture effects. This raises two questions: first, are their results from the training phase replicable? and, second, if so, why did they find these rather untypical effects, that is, which underlying mechanisms and processes may account for it? Answering these questions should be informative with respect to the training-test paradigm used for research on VDAC.

### Underlying Mechanisms and Processes

Sha and Jiang (2016) observed a search benefit for high-reward targets relative to low-reward targets or targets not associated with any reward in their training. In visual search, such search benefits can originate from two sources: first, from an increased efficiency of the search process and, second, from differences in non-search processes (cf. Wolfe, 2016). Examples for the latter are sensory or motor processes (e.g., Yoshimura et al., 2019). Differences in search efficiency are attributed to the extent that attention is *guided* to a stimulus and the extent to which selected stimuli are processed (Wolfe, 2016). The results of previous research (e.g., Lee and Shomstein, 2014) suggest that both search efficiency and non-search processing speed increase with increasing reward. This fits the assumption that color guide’ attention to the target, but that the associated reward modulates this guidance (Wolfe and Horowitz, 2017). For instance, the associated reward might result in an increase of the salience of the target color (cf. Hickey et al., 2010).

While this explains reward-related benefits in general, it does not explain why Sha and Jiang (2016) observed reward-related benefits in the training phase, whereas others did not (e.g., Roper et al., 2014). For some reason, the reward-related effects on performance might have been increased in the study of Sha and Jiang (2016). As already mentioned, the most obvious difference between the study of Sha and Jiang (2016) and other studies lies in the set of line orientations used for target and distractor stimuli. In the former, target and distractor line orientations varied along the same set (i.e., horizontal vs. vertical). Thus, the target circle could only be determined based on its color, but not based on its line orientation since the same orientation was also present in the distractors. In training-test paradigms where target and distractor lines varied within different sets of orientations, however, the target has two unique features (i.e., color and line orientation). Consequently, the task is easier in these studies compared to the one of the study by Sha and Jiang (2016). The diverging results between the studies might therefore be due to different degrees of effort required for performing the tasks (see Kahneman, 1973, for a review about effort and task demands).

From this perspective, two different – but not mutually exclusive – explanations are possible. First, the increased task demand in the study of Sha and Jiang (2016) might have resulted in a decreased search efficiency and, therefore, increased the necessary effort (cf. Porter et al., 2007) to find especially the attentionally less prioritized targets (i.e., those associated with low or no reward). Consequently, the search for targets associated with low or no reward might be slowed. We will refer to this as *search disadvantage* assumption.

Second, since the participants’ aim is to earn money, the reward-related colors might be part of the participants’ current *attentional set* (cf. Folk et al., 1992; Bacon and Egeth, 1994), i.e., their internal goal template, resulting in top-down prioritization of the reward-related colors. This prioritization could even be proportional to the associated reward. The increased task demands in a search task like the one used by Sha and Jiang (2016) might require additional attentional resources. Due to the relevance of the reward association for participants’ goals, these additional resources might serve to further enhance the top-down prioritization.^1^ We will refer to this explanation as *top-down enhancement* assumption.

In sum, the more demanding the search task, the more effort is needed to solve the task, and the larger the difference between stimuli associated with high reward relative to stimuli associated with low or no reward should be. From the perspective of the search disadvantage assumption, this pattern results from less efficient searches for targets associated with low or no reward. From the perspective of the top-down enhancement assumption, it results from the reward-related colors being additionally prioritized top-down in a way proportional to the reward.

### The Present Study

In this study, we specifically focused on the learning of the reward associations, hence we did not include a test phase in our experiments. Our aims were (a) to conceptually replicate the results of the training phase of Sha and Jiang (2016) and (b) to extend their findings in an attempt to examine the underlying mechanisms and processes. Since our main focus was on the latter, we had to change the basic design of Sha and Jiang (2016) to investigate these mechanisms and processes in detail. However, as outlined above, we assumed that the orientation of the line element within the targets and distractors was crucial with respect to their results. Therefore, we used the same line orientations as Sha and Jiang (2016) in a similar study design. More specifically, we used a visual-search task, where participants had to look for a target circle in one out of three reward-associated colors among other differently colored (Experiment 1) or gray (Experiment 2) distractor circles. Participants then had to categorize the line inside the target circle with respect to its orientation. Importantly, line orientation varied on the same horizontal vs. vertical dimension in the target *and* the distractors (cf. Qi et al., 2013; Sha and Jiang, 2016). As will be shown, under these conditions we also observed reward-related search benefits.

To investigate the underlying mechanisms and processes, we analyzed the data in two steps. First, we examined possible reward-related differences in search efficiency and non-search processing speed. Both components can be investigated by examining the search function, where the slope is an indicator for search efficiency and the intercept is an indicator for non-search processing speed (cf. Wolfe, 2016). If the slopes become flatter the higher the reward, this would indicate an increase in search efficiency. If, however, the slopes remain constant, but the offset of the search functions between the reward conditions differ (i.e., faster responses the higher the reward), this would indicate reward-induced changes in non-search processing speed. Based on previous research (e.g., Lee and Shomstein, 2014), we predicted that both should increase with increasing reward.

Second, to investigate whether increased task demands result in greater reward-related benefits due to increased necessary effort, we used a demanding (Experiment 1) and a less demanding search task (Experiment 2) and examined how the reward-related benefits develop over time, i.e., how they are present in different portions of the response time (RT) distribution. Since there is evidence that required effort increases as the duration of the search process increases (cf. Porter et al., 2007), we assumed that a modulation of reward-related effects due to task demands might be especially visible in longer RTs. The change in reward-related capture effects over time was only investigated in a few studies so far. Failing et al. (Failing et al., 2015; see also Preciado and Theeuwes, 2018), for instance, showed that the proportion of first saccades (i.e., rapid eye movements between two fixation points, cf. Holmqvist et al., 2011) directed to a reward-related distractor was highest, if the corresponding saccade was executed early after the stimulus display appeared. However, the proportion of first saccades directed to the reward-related distractor declined the longer it took to execute the saccade. The authors argued that the reward association results in oculomotor capture by the corresponding stimulus and that it influences attentional selection on early processing stages (see also, e.g., Anderson et al., 2011a). They further assumed that the reward-related stimuli are suppressed top-down on later processing stages, explaining the reduction of reward effects over time. Since in our study the reward association was manipulated within the target, we considered a top-down suppression of the reward-related stimulus as unlikely. From this perspective, it is conceivable that with a more demanding task (as in section Experiment 1), the influence of reward-related effects might be the same, irrespective of RTs. If, however, the reward-related effects increase with RT, this can be seen as evidence in favor of our assumption that effort plays a crucial role in reward-related attentional prioritization. Or, in other words, that additional (top-down) processes play a crucial role besides pure attentional capture in respective tasks.

To anticipate our results, with a demanding task (Experiment 1), we found that reward-related effects are modulated by RT, i.e., they increase as RT increases. Using a less demanding task in Experiment 2, we examined this time-dependent modulation of the reward-related effect in more detail by trying to disentangle the search disadvantage and top-down enhancement assumptions outlined above. To this aim, we used a search task where each target was the only colored stimulus in the display and, thus, it should pop out (cf. Treisman, 1985) irrespective of the reward condition. Clearly, this task is less demanding, hence less effort is needed to find the target. As a consequence, there should not be a search disadvantage for colors associated with low or no reward. Therefore, from the perspective of the search disadvantage assumption, there should not be an increase in the reward-related effects with RT. If, however, the reward-associated colors are nevertheless additionally prioritized top-down, effects of this prioritization should also be visible in a less demanding task (albeit possibly reduced). Thus, in this case, an increase in the reward-related benefits with increasing RTs should be observed again. As will be shown, in Experiment 2, we found evidence for the search disadvantage assumption but not for the top-down enhancement assumption.

## Experiment 1

We used a visual search task, in which participants saw either two or eight colored circles, each containing either a horizontal or a vertical line (cf. Qi et al., 2013; Sha and Jiang, 2016). The participants’ task was to indicate the line orientation within the circle colored in one out of three target colors. Each of these colors was associated with either high, low, or no reward. To investigate whether the predicted reward-related benefits in search speed are due to increased search efficiency or non-search processing speed, differently from Sha and Jiang (2016), we used two set-sizes, similar to Lee and Shomstein (2014).

Since reward-related performance benefits were also found if participants had to respond within an individually adjusted deadline (cf. Kiss et al., 2009; Müller et al., 2016), we assumed that time pressure might amplify reward-related performance benefits by increasing task demands and, consequently, participants’ effort (cf. Kahneman, 1973). Thus, we set an individually adjusted performance-related deadline after the first half of the experiment as an additional within-experiment manipulation of task demand. Since Sha and Jiang (2016) did not use such a deadline procedure, we used the deadline only in the second half of the experiment, to be able to more directly compare our results of the first half of the experiment with their results.

### Materials and Methods

#### Participants

Twenty four participants (17 female) recruited *via* the online recruitment tool (SONA) of the University of Konstanz attended the Experiment in exchange for a variable payment of up to 18 € maximum (2 € base payment, up to 16 € performance-dependent payment; on average participants earned 16.12 €). Their age ranged from 21 to 42 years (*M*_age_ = 24.92, *SD*_age_ = 4.20), and all reported normal or corrected-to-normal vision. Informed consent in line with the 1964 Declaration of Helsinki and its later amendments, as well as in agreement with the ethics and safety guidelines at the University of Konstanz was obtained from all participants *via* check-marking an according box on the informed-consent instruction page before the actual experiment started. Participants were informed that they are free to withdraw from the study at any point in time without any negative repercussions.

#### Apparatus

Participants were tested in groups of up to 10 in a group lab. Stimulus presentation and response recording were controlled by PCs. The stimuli were presented on 23.8-inch color monitors (Fujitsu B248T) with a resolution of 1,920 × 1,080 pixels and a refresh rate of 60 Hz. The screen was located centrally on a desk in front of the participants, with a viewing distance of about 60 cm. The experiment was programed in JavaScript and ran under Google Chrome (Versions 68–70) in a Windows 10 environment. Responses had to be entered by pressing the Y- or M-button on a standard German QWERTZ keyboard.

#### Stimuli and Task

**Figure 1:**
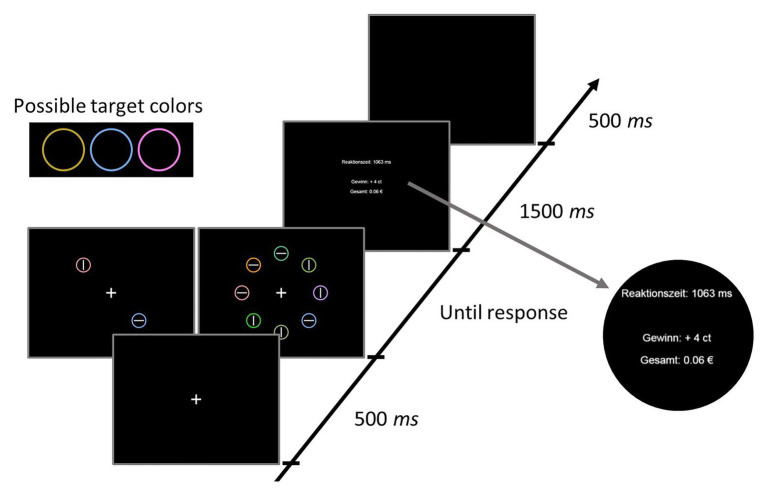
Stimuli and timing of events in one trial of Experiment 1. Reaktionszeit = response time; Gewinn = reward earned in current trial; Gesamt = overall reward.

On each trial, a fixation display, a search display, and a feedback display were presented (see Figure 1). The fixation display contained a fixation cross presented in white (RGB: 255, 255, 255) on a black (RGB: 0, 0, 0) background at the center of the screen. The fixation cross was about 1.31° visual angle in width and height. The search display consisted of either two or eight colored circles, each containing either a horizontal or a vertical line in the center. In the condition with eight circles (set-size 8), the circles were placed at equal distances along an imaginary circle with a radius of about 5.24° visual angle around the screen center. The radius of each individual colored circle was about 0.92° visual angle, and the lengths of the lines within the circles were about 1.31° visual angle in width or height (depending on orientation; thickness about 0.16°). In the condition with the two circles (set-size 2), they were placed opposite of each other on the imaginary circle, occupying two of the eight possible positions from the set-size 8 (see Figure 1). The targets were colored either in yellow (RGB: 186, 158, 34), blue (RGB: 107, 162, 227), or pink (RGB: 254, 118, 230). Only one of the target colors was present on a given trial. The colors for the distractors were selected randomly from the set of violet (RGB: 189, 135, 253), dark beige (RGB: 224, 145, 51), orange (RGB: 251, 141, 11), light olive (RGB: 138,165,106), green (RGB: 121,175,57), turquois (RGB: 73,182,129), and bright green (RGB: 39,202,31), with the restriction that each color could only appear once within a search display. The lines within the colored circles were always white (RGB: 255, 255, 255). Participants’ task was to locate the circle in one of the target colors, and then decide as quickly as possible which orientation the line had (horizontal or vertical) by pressing either the Y- or the M-key. The orientation-to-key mapping was counterbalanced across participants.

After responding, the search display was removed and feedback was presented. The feedback contained information about the RT in the current trial, the monetary reward achieved in the current trial (see below for further details), and the overall money earned so far during the experiment (see Figure 1).

#### Procedure

Each trial consisted of the following sequence of events (see Figure 1): First, a fixation cross was presented at the center of the screen for 500 ms. Then the stimulus array appeared additionally to the fixation cross and remained on screen until a response was given. After responding, participants received acoustic feedback (a tone presented for about 100 ms *via* headphone) in case they made an error, and visual feedback about their performance for 1,500 ms. The screen turned black again for 500 ms, before the next trial started.

Before the actual experiment, participants performed two practice blocks with 96 trials each, where they could not yet gain any money, and which were not analyzed. The first of these practice blocks served to familiarize the participants with the target colors, hence target stimuli were always colored in one of the three colors, whereas the distractors were always gray (RGB: 155, 155, 155). In the second practice block, the distractors were colored as in the actual test blocks. Overall, participants ran through 10 test blocks with 96 trials each. After each block, participants received summarized feedback about their performance in that block (i.e., mean RT, error rate, and overall monetary gain so far).

The test blocks were separated into two groups: In the first five blocks, participants performed the search task without time limit. In blocks 6–10, participants were given a set-size specific response deadline and were informed that from now on they only receive their respective reward if they respond correctly and within the time limit. The deadline was determined as the set-size specific median RT of all trials from the last block without deadline (i.e., Block 5). These deadlines remained unchanged for the remainder of the experiment.

On each trial, possible reward depended on the target color. One color was associated with a gain of 0 Eurocent (no reward), a second color with a gain of 1 Eurocent (low reward), and the third color with a gain of 4 Eurocent (high reward). The association of reward to color was nonprobabilistic, i.e., participants received the respective reward on all trials (if they responded correctly and, in blocks 6–10, within the time limit). Each color appeared equally often throughout the experiment and within each block of trials. The order of appearance of the colors within each block was randomized. The color-to-reward mapping was counterbalanced across participants.

### Results

The data were analyzed using R (Version 3.6.0; R Core Team, 2019) and RStudio (Version 1.2.1335, R Studio Team, 2018). For all analyses, Greenhouse-Geisser-corrected degrees of freedom and values of *p* were reported, where the assumption of sphericity was violated (Greenhouse and Geisser, 1959). Outliers were eliminated by excluding trials with RTs < 100 ms and RTs > *M*_RT_ + 3 * *SD*_RT_ separately for each participant and cell of the design from the overall data, where *M*_RT_ and *SD*_RT_ were calculated from correct trials. This resulted in an exclusion of 1.42% of the overall data. The accuracy in the remaining trials was 91.92%. Only correct trials were considered in the RT analyses. In cases, in which several *post-hoc* comparisons were required, we adjusted the significance criterion by using the Bonferroni correction (cf. VanderWeele and Mathur, 2019). For transparency, we will report the Bonferroni-adjusted significance criterion, where the *post hoc* test would be significant, if unadjusted. We reported partial eta square (*η*_p_^2^) for ANOVA results and Cohen’s *d* for the paired *t*-tests (*d*_z_; cf. Lakens, 2013).

For the analyses, we included all trials in the condition with time pressure irrespective of timeout errors for two reasons: First, we did not present an overt deadline signal to the participants, indicating that their time was up. Thus, at the moment they actually pressed the response key, they were most likely unaware of whether they had met the deadline or not (cf. Stankevich and Geng, 2014). Second, removing trials from the deadline condition, but not from the no-deadline condition would have resulted in the data reflecting different portions of the overall RT distributions. This would have compromised any direct comparisons and, therefore, was considered inadequate.

#### Overall Analyses

To determine the effects of reward on search efficiency and non-search processing speed, three-way ANOVAs with repeated measures on the factors deadline (no and yes), set-size (2 and 8), and reward (no, low, and high) were computed. *Post-hoc t*-tests were performed to determine differences between reward conditions, where this factor revealed a significant main effect or entered a significant interaction.

##### Response Times

**Figure 2:**
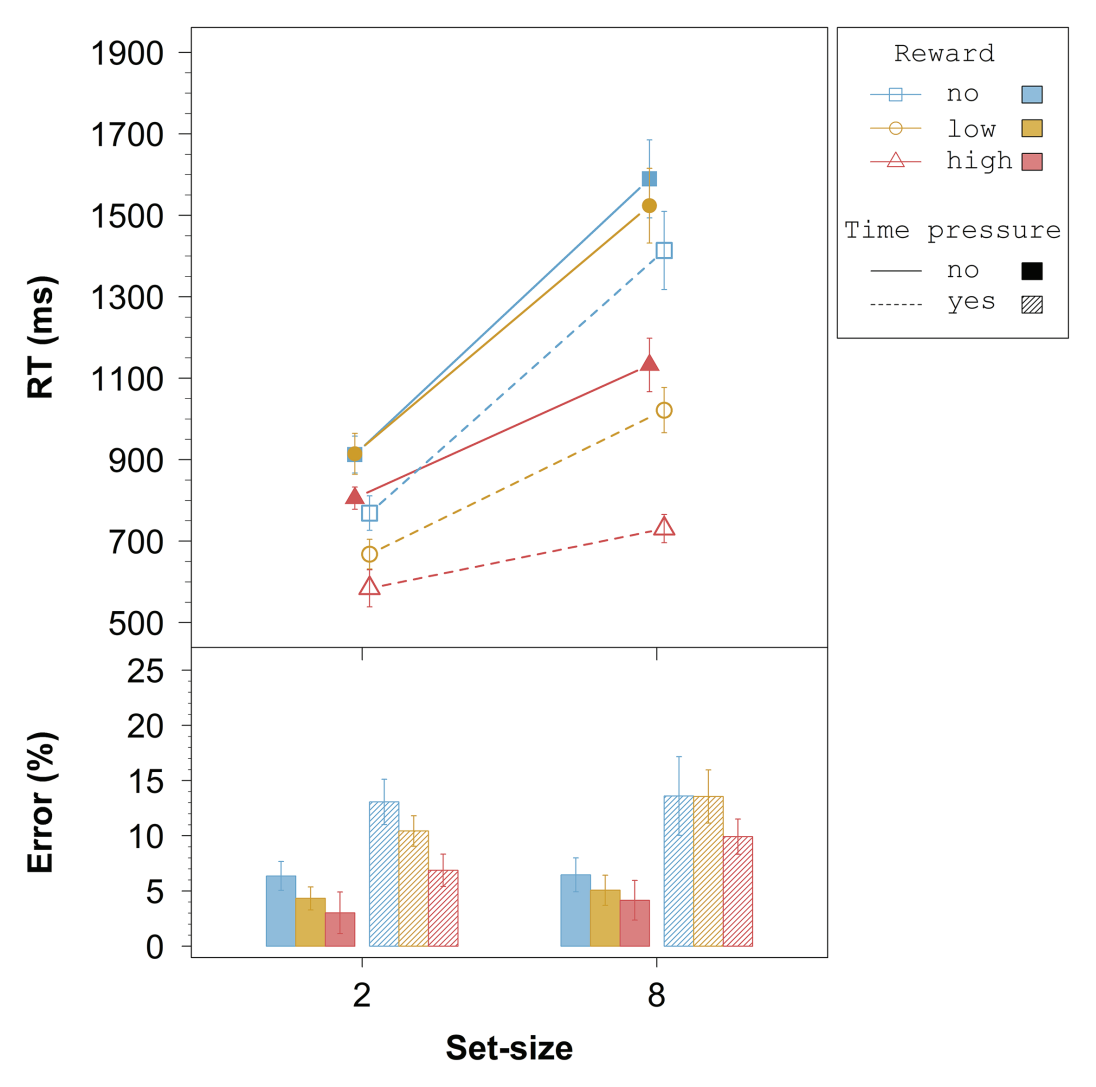
Averaged overall data from Experiment 1. Error bars represent 95% within-subject confidence intervals (Loftus and Masson, 1994; Morey, 2008). RT = response time.

The results of the overall ANOVA for the RTs are summarized in Table 1. All main effects and interactions were significant. Figure 2 (upper panel) shows that without time pressure (solid lines), across set-size there was no difference between no reward and low reward, *t*(23) = 0.57, *p* = 0.58, *d_z_* = 0.12, whereas high reward differed significantly from both, no reward, *t*(23) = 5.34, *p* < 0.001, *d_z_* = 1.09, and low reward, *t*(23) = 7.58, *p* < 0.001, *d_z_* = 1.55. With time pressure (dashed lines), all differences between reward conditions were significant: no vs. low, *t*(23) = 6.06, *p* < 0.001, *d_z_* = 1.24, no vs. high, *t*(23) = 10.50, *p* < 0.001, *d_z_* = 2.14, and low vs. high, *t*(23) = 9.09, *p* < 0.001, *d_z_* = 1.86.

**Table 1:** Results of the overall ANOVA for the response times in Experiment 1.

Predictor	Df_N_	Df_D_	ε	*F*	*p*	*η_p_^2^*[95% CI]
Deadline (Dl)	1	23	-	38.28	<0.001	0.62 [0.33, 0.76]
Set-size (SetS)	1	23	-	213.07	<0.001	0.90 [0.80, 0.94]
Reward (Rew)	1.36	31.25	0.68	58.93	<0.001	0.72 [0.55, 0.79]
Dl * SetS	1	23	-	28.63	<0.001	0.55 [0.24, 0.71]
Dl * Rew	1.59	36.54	0.79	8.69	0.002	0.27 [0.06, 0.44]
SetS * Rew	1.37	31.40	0.68	59.10	<0.001	0.72 [0.56, 0.80]
Dl * SetS * Rew	1.46	33.52	0.73	5.97	0.011	0.21 [0.02, 0.37]

As indicated by the significant three-way interaction in the overall ANOVA, the RT-differences between no reward and low reward in the no time pressure condition did not differ significantly between set-size 2 and set-size 8, *t*(23) = −0.93, *p* = 0.36, *d_z_* = 0.19, whereas the RT-differences between no reward and high reward, *t*(23) = −5.32, *p* < 0.001, *d_z_* = 1.09, and between low reward and high reward, *t*(23) = −7.46, *p* < 0.001, *d_z_* = 1.52, differed significantly between set-sizes (see Figure 2). In the condition with time pressure, all the RT-differences between reward conditions differed significantly between set-sizes: no vs. low, *t*(23) = −6.49, *p* < 0.001, *d_z_* = 1.32; no vs. high, *t*(23) = −10.75, *p* < 0.001, *d_z_* = 2.19; low vs. high, *t*(23) = −9.75, *p* < 0.001, *d_z_* = 1.99.

##### Error Rates

The results of the overall ANOVA for the error rates are summarized in Table 2. Only the three main effects were significant. Follow-up *t*-tests for the effect of reward revealed marginally significant overall differences between no reward and low reward, *t*(23) = 1.81, *p* = 0.083, *d_z_* = 0.37, between low reward and high reward, *t*(23) = 1.82, *p* = 0.082, *d_z_* = 0.37, and between no reward and high reward, *t*(23) = 2.34, *p* = 0.029, *d_z_* = 0.48 (with a Bonferroni-adjusted alpha of 0.0167). From Figure 2 (bottom panel), it can be seen that the higher the reward the lower the error rate, irrespective of time pressure and set-size. Moreover, the error rate was slightly higher for the set-size 8 than for the set-size 2 condition, and considerably higher with time pressure than without time pressure.

**Table 2:** Results of the overall ANOVA for the error rates in Experiment 1.

Predictor	Df_N_	Df_D_	*ε*	*F*	*p*	η_p_^2^ [95% CI]
Deadline (Dl)	1	23	-	24.57	<0.001	0.52 [0.20, 0.68]
Set-size (SetS)	1	23	-	5.29	0.031	0.19 [0.00, 0.43]
Reward (Rew)	1.35	31.16	0.68	4.46	0.032	0.16 [0.005, 0.33]
Dl * SetS	1	23	-	2.09	0.16	0.08 [0.00, 0.32]
Dl * Rew	2	46	-	1.62	0.21	0.07 [0.00, 0.21]
SetS * Rew	1.58	36.45	0.79	1.59	0.22	0.06 [0.00, 0.21]
Dl * SetS * Rew	1.52	34.92	0.76	0.44	0.60	0.02 [0.00, 0.12]

#### Cumulative Distribution Functions and Distributional Analyses

**Figure 3:**
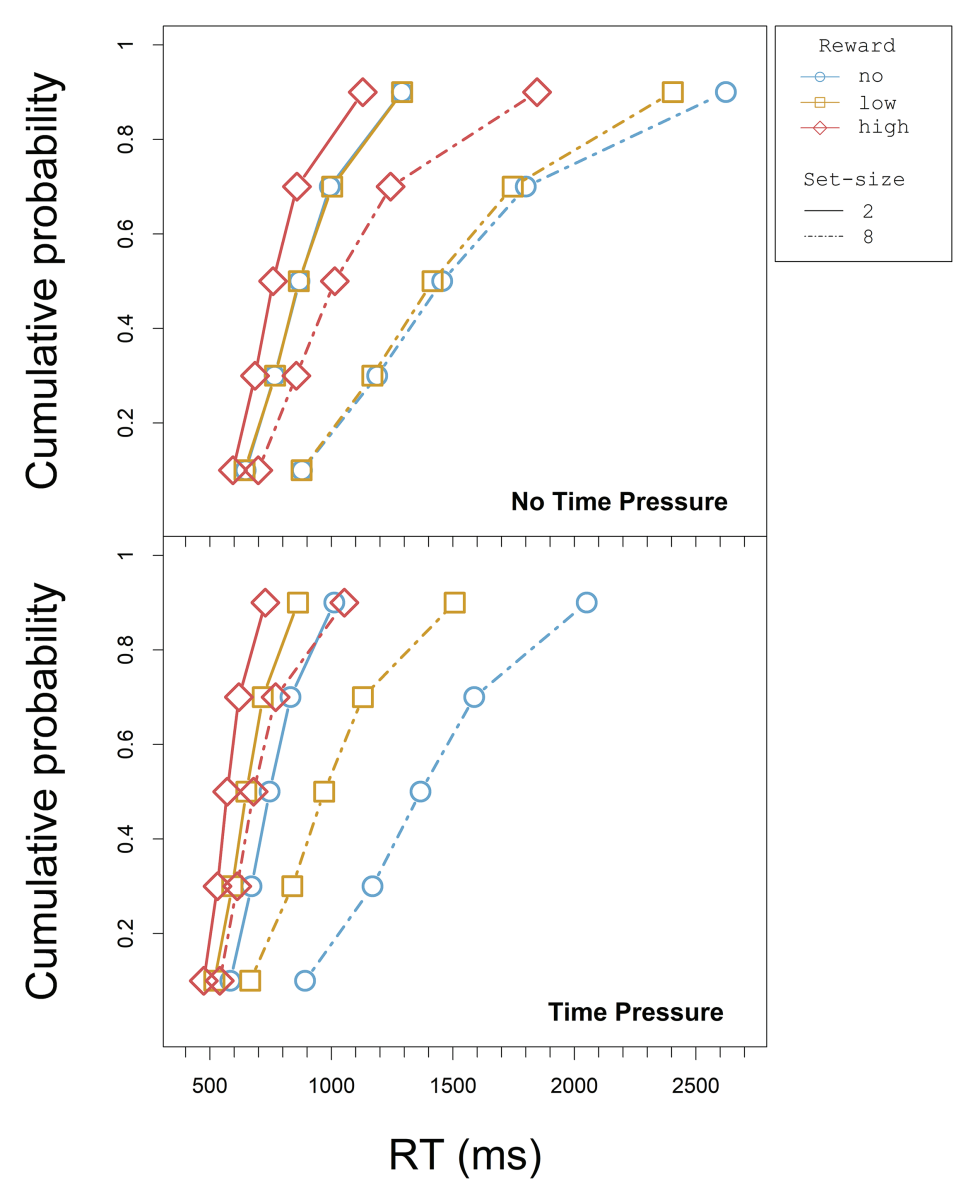
CDFs for each set-size and reward condition without and with time pressure in Experiment 1.

To investigate the time-dependence of the reward effects in the RTs, we looked at the cumulative distribution functions (CDFs) for the different reward conditions and at the *relative* reward effect across the different vincentiles (see below). We used Vincent averaging (Vincent, 1912; Ratcliff, 1979, see also Andrews and Heathcote, 2001) to accumulate the distributional data. That is, we ordered each participant’s RT in an increasing order and determined the quintiles of the RT distribution for each design cell (reward, set-size, and deadline, separately for each participant). The values within each individual quintile were averaged for each condition to build *vincentiles* (cf. Andrews and Heathcote, 2001). To construct the CDFs, the vincentiles in turn were averaged across participants for each condition and quintile. The CDFs for all set-size and reward conditions are displayed separately for the blocks with and without time pressure in Figure 3.

To exclude that possible reward effects might only reflect scaling (i.e., increasing effects due to increasing RT), we examined the relative reward effect for the different vincentiles of each set size and time pressure condition. Thus, instead of simply averaging the vincentiles across participants and conditions, we averaged the relative reward effect. The relative reward effect for each design cell was calculated by subtracting each high-reward vincentile from the respective low-reward vincentile and dividing this difference by the combined mean RTs of these high- and low- reward vincentiles. We used the high- and low- reward conditions here as points of reference, since differential influences of both conditions on attention could only be traced back to the difference in associated reward (cf. Watson et al., 2019). Positive relative reward effects correspond to performance benefits for the high- reward condition relative to the low-reward condition (i.e., participants were faster), while negative effects correspond to disadvantages (i.e., participants were slower).

**Figure 4:**
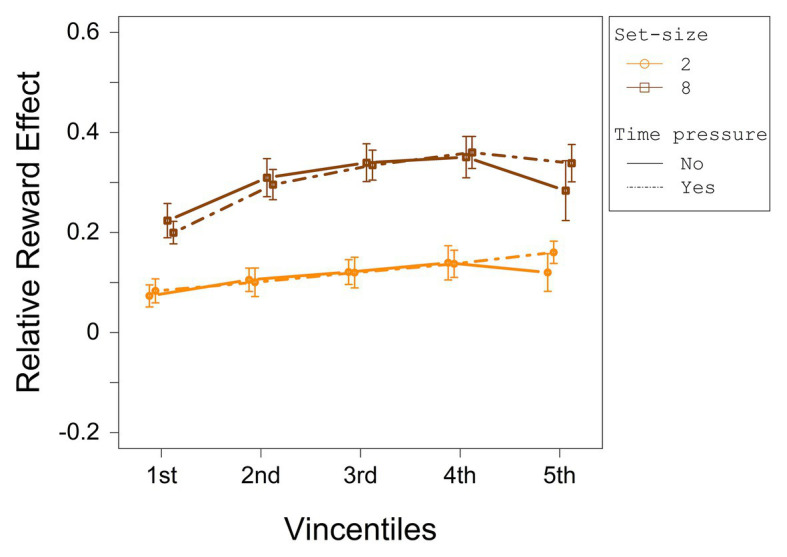
The relative reward effect depending on set-size and deadline for each vincentile in Experiment 1. Error bars represent 95% within-subject confidence intervals (Loftus and Masson, 1994; Morey, 2008).

To investigate the distributional data of the relative reward effect statistically, a three-way ANOVA with repeated measures on the factors set-size (2 and 8), deadline (no deadline and deadline), and vincentile (1, 2, 3, 4, and 5) was computed with the relative reward effect as dependent variable. The relative reward effect depending on set-size and deadline condition for each vincentile is displayed in Figure 4. The results of the three-way ANOVA for the relative reward effect are summarized in Table 3.

**Table 3:** Results of the distributional analysis of the relative reward effect in Experiment 1.

Predictor	Df_N_	Df_D_	*ε*	*F*	*p*	η_p_^2^ [95% CI]
Deadline (Dl)	1	23	-	0.05	0.83	<0.01 [0.00,0.13]
Set-size (SetS)	1	23	-	118.01	<0.001	0.84 [0.67,0.89]
Vincentile (Vin)	1.43	32.88	0.36	21.77	<0.001	0.49 [0.32,0.58]
Dl * SetS	1	23	-	0.03	0.86	<0.01 [0.00,0.12]
Dl * Vin	1.72	39.47	0.43	2.04	0.15	0.08 [0.00,0.17]
SetS * Vin	1.84	42.41	0.46	11.53	<0.001	0.33 [0.16,0.44]
Dl* SetS * Vin	1.66	38.21	0.42	0.84	0.42	0.04 [0.00,0.09]

The analysis revealed a significant main effect for set-size and vincentile. Moreover, set-size and vincentile interacted significantly. The main effect of set-size indicates that the relative difference between high reward and low reward increased with set-size (see overall ANOVA for significance tests). Figure 4 shows that the relative reward effect increased from the first to the second vincentile, *t*(23) = −9.65, *p* = <0.001, *d_z_* = 1.97, from the second to the third vincentile, *t*(23) = −5.64, *p* = <0.001, *d_z_* = 1.15, and from the third to the fourth vincentile, *t*(23) = −3.29, *p* = 0.003, *d_z_* = 0.67. The relative reward effect did not differ between the fourth and the fifth vincentile, *t*(23) = 1.68, *p* = 0.11, *d_z_* = 0.34.

Considering the interaction between set-size and vincentile, in the set-size 2 condition, the relative reward effect increased from the first to the second vincentile *t*(23) = −4.48, *p* = <0.001, *d_z_* = 0.92, from the second to the third vincentile, *t*(23) = −5.14, *p* = <0.001, *d_z_* = 1.05, and from the third to the fourth vincentile, *t*(23) = −3.27, *p* = 0.003, *d_z_* = 0.67. The reward effect did not differ between the fourth and the fifth vincentile, *t*(23) = −0.15, *p* = 0.88, *d_z_* = 0.03. In the set-size 8 condition, the relative reward effect increased from the first to the second vincentile, *t*(23) = −9.95, *p* = <0.001, *d_z_* = 2.03 and from the second to the third vincentile, *t*(23) = −4.31, *p* = <0.001, *d_z_* = 0.88. The differences between the third and the fourth vincentile as well as between the fourth and the fifth vincentile were only marginally significant (with a Bonferroni-adjusted alpha of 0.0063), *t*(23) = −2.15, *p* = 0.042, *d_z_* = 0.44, and *t*(23) = 2.68, *p* = 0.013, *d_z_* = 0.55, respectively.

### Discussion

Without time pressure, we observed search benefits for the high-reward condition relative to the low- and no-reward one. The low-reward condition, though, was inseparable from the no-reward condition. But with time pressure, there was a search benefit for low- and high-reward stimuli relative to no-reward stimuli. Thus, our results conceptually replicate the results of Sha and Jiang (2016). But how can the results be explained from a more mechanistic point of view?

The overall analysis provides evidence that the influence of reward on performance might be due to increases in search efficiency and in non-search processing speed. First, without time pressure, but even more so with time pressure, search slopes decreased depending on the associated reward, indicating that targets pop-out the more the higher the associated reward. This result supports the assumption that reward-associated stimuli become perceptually more salient over time (e.g., Hickey et al., 2010), resulting in increased search efficiency. Second, the reward search functions also differed in their intercepts, indicating that non-search processing speed increases the higher the associated reward. Since it was shown that differences in salience result in intercept changes if search efficiency is already nearly optimal (e.g., Zehetleitner and Müller, 2010), these intercept differences are an additional hint that the associated reward influences salience (Hickey et al., 2010).

The analysis of the relative reward effect showed that high reward influenced performance already in the fastest responses, *t*(23) = 10.95, *p* < 0.001, *d* = 2.23, which is in line with other studies, and suggests that reward-associated stimuli capture attention (cf. Failing and Theeuwes, 2018). The effect increased throughout the first four vincentiles in the set-size 2 condition and throughout the first three vincentiles in the set-size 8 condition. Moreover, the relative reward effect was larger for set-size 8 relative to set-size 2. These results indicate that the longer the search process takes, the larger the reward-related effect on performance gets, even if possible scaling effects are accounted for. We will come back to this point in Experiment 2.

## Experiment 2

In Experiment 1, the relative reward effect increased over the first three vincentiles in the set-size 8 condition and over the first four vincentiles in the set-size 2 condition, even if we controlled for scaling effects. This indicates that the longer participants have to search for the target, and the more effort it takes, the stronger the reward effect. A possible explanation is that the target color is additionally prioritized top-down depending on the associated reward (cf. top-down enhancement assumption). In this case, we should also observe an increase in the relative reward effect with RT in a less demanding task. Alternatively, in demanding search tasks, participants have to put more effort in the search for targets associated with less or no reward, resulting in a search disadvantage for these colors (cf. search disadvantage assumption). This explanation would be supported, if a reduction in demand results in stable reward effects with increasing RTs. Note that both explanations are not mutually exclusive. In Experiment 2, we tried to disentangle both explanations by reducing the demands on the search process.

Our second experiment was similar to the first one, except that we used a “pop-out” search task. Specifically, the distractor circles were always gray, so that the colored target circle popped out from the array. This manipulation should result in flat search slopes (cf. Wolfe, 2016). Thus, reward-related differences in search efficiency should be eliminated, but differences in non-search processes should remain. In line with this assumption, by using a similar design, Lee and Shomstein (2014) observed (a) reward-related search benefits for high reward vs. low reward without time pressure (see Kiss et al., 2009, for a similar result with time pressure) and (b) flat search slopes for both reward conditions. However, neither of these studies examined the dependence of the reward-related effect on response speed.

### Materials and Methods

#### Participants

Twenty four new participants (18 female) recruited *via* the online recruitment tool (SONA) of the University of Konstanz took part in the Experiment in exchange for a variable payment of up to 18 € maximum (2 € base payment, 16 € performance-dependent payment; on average participants earned 15.58 €). Their age ranged from 19 to 42 years (*M*_age_ = 24.08, *SD*_age_ = 5.03). All other recruitment and participation criteria were the same as in Experiment 1.

#### Apparatus, Stimuli, and Procedure

**Figure 5:**
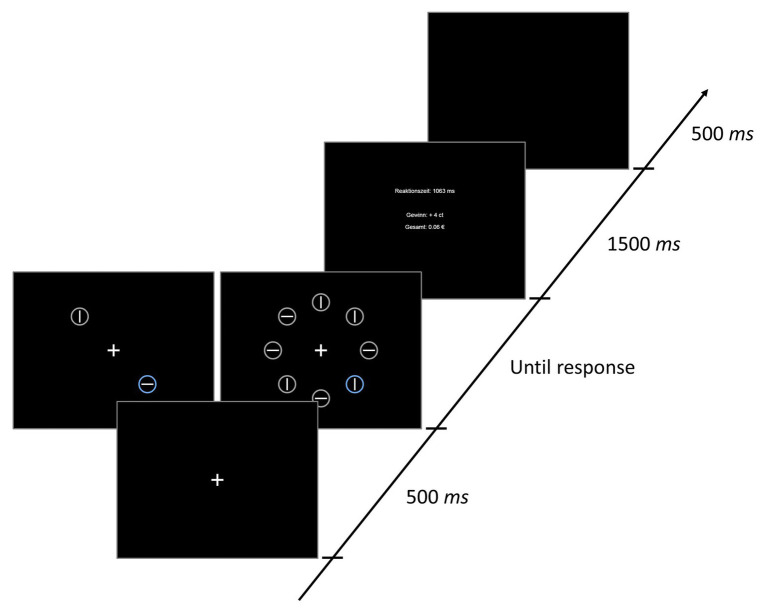
Stimuli (example) and timing of events in Experiment 2.

The apparatus, stimuli, and procedure were the same as in Experiment 1, except that the distractors were now always gray (RGB: 155, 155, 155; see Figure 5), and only the target appeared in one out of the three pre-defined colors (see Figure 1).

### Results

We used the same trim procedure and analyses as in Experiment 1. The trim procedure resulted in a removal of 1.40% of the data. The accuracy in the remaining data was 90.10%.

#### Overall Analyses

##### Response Times

**Figure 6:**
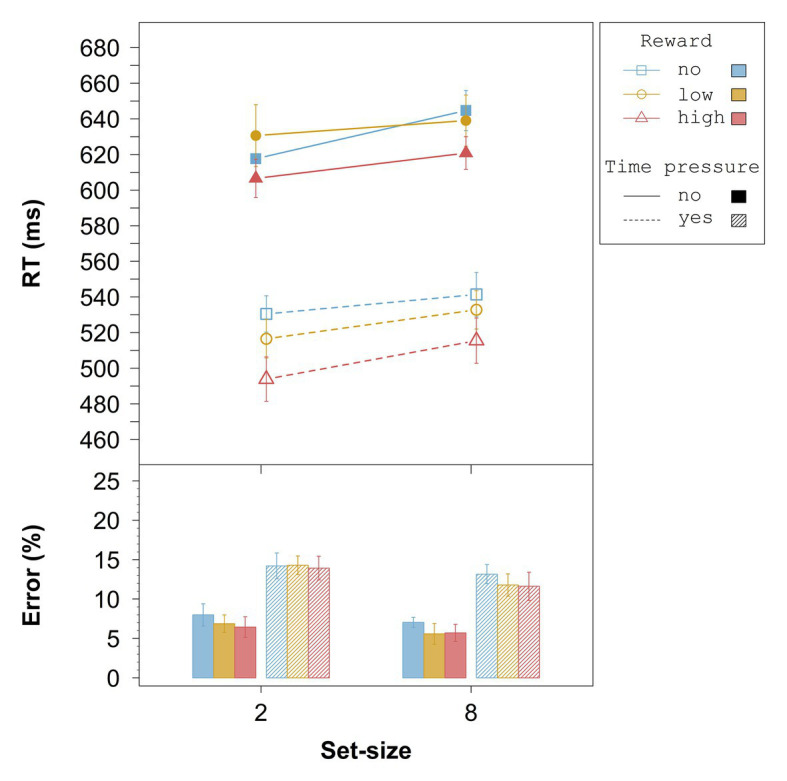
Averaged overall data from Experiment 2. Error bars represent 95% within-subject confidence intervals (Loftus and Masson, 1994; Morey, 2008). RT = response time.

The results of the overall ANOVA for the RTs are summarized in Table 4. All three main effects were significant. Moreover, the two-way interaction between deadline and reward, and the three-way interaction were also significant. Figure 6 (upper panel) shows that without time pressure (solid lines), across set-size there was no difference between no reward and low reward, *t*(23) = −0.49, *p* = 0.63, *d_z_* = 0.10, whereas high reward differed marginally significantly from both no reward, *t*(23) = −2.85, *p* = 0.009, *d_z_* = 0.58, and low reward, *t*(23) = −2.80, *p* = 0.010, *d_z_* = 0.57 (with a Bonferroni-corrected alpha of 0.0083). With time pressure (dashed lines), the difference between no reward and low reward was marginally significant, *t*(23) = 1.97, *p* = 0.062, *d_z_* = 0.40, and the differences between high reward and no reward, *t*(23) = −4.78, *p* < 0.001, *d_z_* = 0.98, as well as between high reward and low reward, *t*(23) = −4.49, *p* < 0.001, *d_z_* = 0.92, were significant.

**Table 4:** Results of the overall ANOVA for the RTs in Experiment 2.

Predictor	Df_N_	Df_D_	*ε*	*F*	*p*	η_p_^2^ [95% CI]
Deadline (Dl)	1	23	-	62.33	<0.001	0.73 [0.49, 0.82]
Set-size (SetS)	1	23	-	49.97	<0.001	0.68 [0.41, 0.80]
Reward (Rew)	2	46	-	11.02	<0.001	0.32 [0.10, 0.48]
Dl * SetS	1	23	-	0.02	0.90	<0.01 [0.00, 0.10]
Dl * Rew	2	46	-	3.58	0.036	0.13 [0.00, 0.30]
SetS * Rew	2	46	-	0.99	0.38	0.04 [0.00, 0.17]
Dl * SetS * Rew	2	46	-	5.58	0.007	0.20 [0.02, 0.36]

To examine the significant three-way interaction, we compared the RT-differences for all pairs of reward conditions between the two set-sizes separately within each time pressure condition. Focusing on the condition without time pressure, this revealed a marginally significant effect in the RT-difference between the no- and low-reward conditions between set-size 2 and set-size 8, *t*(23) = −2.70, *p* = 0.013, *d_z_* = 0.55 (with a Bonferroni-adjusted alpha of 0.0083). Moreover, the difference between no and high reward differed marginally significantly between set-sizes, *t*(23) = −1.83, *p* = 0.081, *d_z_* = 0.37, while the difference between low and high reward did not differ between set-sizes, *t*(23) = 0.62, *p* = 0.54, *d_z_* = 0.13. With time pressure, the RT-differences for no vs. high reward differed marginally significantly between set-size 2 and set-size 8, *t*(23) = 2.17, *p* = 0.040, *d_z_* = 0.44 (with a Bonferroni-adjusted alpha of 0.0083). The RT-differences for no vs. low reward, *t*(23) = 0.95, *p* = 0.35, *d_z_* = 0.19, as well as the differences between low and high reward, *t*(23) = 1.47, *p* = 0.16, *d_z_* = 0.30, did not differ between set-sizes.

##### Error Rates

The results of the overall ANOVA for the error rates are summarized in Table 5. Only the main effects of deadline and set-size were significant, indicating a generally higher error rate under time pressure and with the lower set-size than without time pressure and the higher set-size, respectively (see Figure 6, bottom panel).

**Table 5:** Results of the overall ANOVA for the error rates in Experiment 2.

Predictor	Df_N_	Df_D_	*ε*	*F*	*p*	η_p_^2^ [95% CI]
Deadline (Dl)	1	23	-	33.66	<0.001	0.59 [0.29, 0.74]
Set-size (SetS)	1	23	-	13.89	0.001	0.38 [0.08, 0.58]
Reward (Rew)	1.48	34.05	0.74	1.80	0.19	0.07 [0.00, 0.22]
Dl * SetS	1	23	-	2.01	0.17	0.08 [0.00, 0.32]
Dl * Rew	2	46	-	0.24	0.79	0.01 [0.00, 0.09]
SetS * Rew	2	46	-	0.58	0.57	0.02 [0.00, 0.13]
Dl * SetS * Rew	2	46	-	0.30	0.75	0.01 [0.00, 0.10]

#### Cumulative Distribution Functions and Distributional Analyses

**Figure 7:**
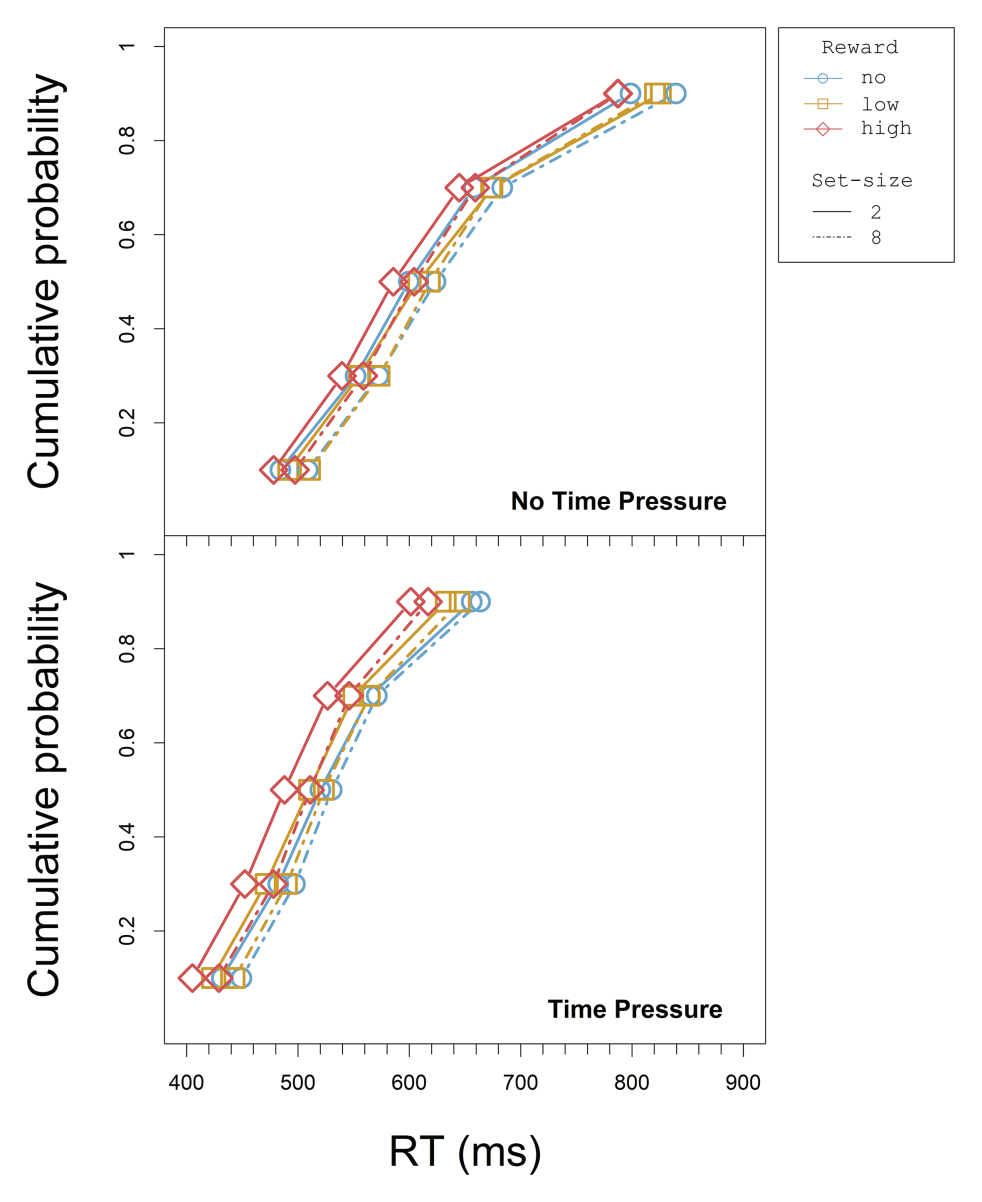
CDFs for each set-size and reward condition without and with time pressure in Experiment 2 (note that the scale of the x axis is not the same as in Figure 3).

The CDFs for all set-size and reward conditions are displayed separately for the blocks with and without time pressure in Figure 7.

**Figure 8:**
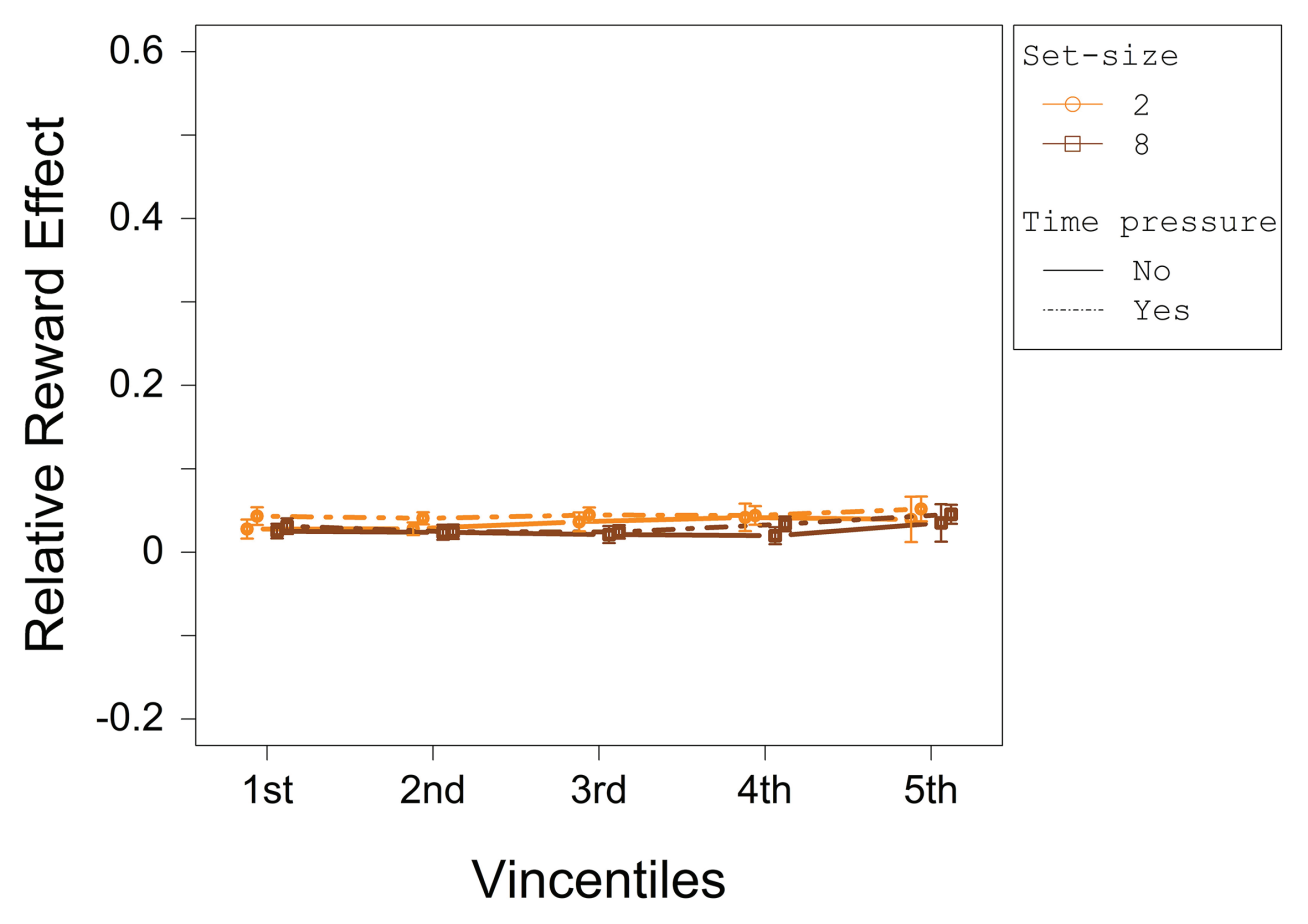
The relative reward effect depending on set-size and deadline for each vincentile in Experiment 2. Error bars represent 95% within-subject confidence intervals (Loftus and Masson, 1994; Morey, 2008).

The relative reward effect depending on set-size and deadline condition for each vincentile is displayed in Figure 8. The results of the three-way ANOVA for the relative reward effect are summarized in Table 6. No main effect or interaction was significant.

**Table 6:** Results of the distributional analysis of the relative reward effect in Experiment 2.

Predictor	Df_N_	Df_D_	*ε*	*F*	*p*	η_p_^2^ [95% CI]
Deadline (Dl)	1	23	-	1.66	0.21	0.07 [0.00, 0.30]
Set-size (SetS)	1	23	-	2.39	0.14	0.09 [0.00, 0.33]
Vincentile (Vin)	1.92	44.07	0.48	2.54	0.092	0.10 [0.00, 0.19]
Dl * SetS	1	23	-	0.08	0.78	<0.01 [0.00, 0.15]
Dl * Vin	1.80	41.44	0.45	0.11	0.88	<0.01 [0.00, 1.00]
SetS * Vin	1.60	36.91	0.40	0.61	0.51	0.03 [0.00, 0.07]
Dl* SetS * Vin	1.99	45.86	0.50	0.55	0.58	0.02 [0.00, 0.07]

#### Common Distributional Analysis of Experiment 1 and Experiment 2

In Experiment 1, the relative reward effect differed significantly between vincentiles, whereas in Experiment 2, it did not. To investigate whether this effect differed significantly between both experiments, we conducted an ANOVA with the factors experiment (first and second) and vincentiles (1, 2, 3, 4, and 5). The results are displayed in Table 7. Both main effects and, importantly, the interaction were significant, reflecting that the relative reward effect differed between vincentiles in Experiment 1, but not in Experiment 2.

**Table 7:** Results of the common distributional analysis of the relative reward effect in Experiments 1 and 2.

Predictor	Df_N_	Df_D_	*ε*	*F*	*p*	η_p_^2^ [95% CI]
Experiment (Exp)	1	46	-	111.90	<0.001	0.71 [0.55, 0.79]
Vincentiles (Vin)	1.49	68.76	0.37	21.02	<0.001	0.31 [0.20, 0.40]
Exp * Vin	1.49	68.76	0.37	17.36	<0.001	0.27 [0.16, 0.36]

### Discussion

In a less demanding pop-out search task, high-reward targets were only found faster than no- or low-reward targets if there was additional time pressure. Contrary to Experiment 1, though, low reward was inseparable from no reward in both time pressure conditions. These results are in line with our assumption that the influence of reward-related performance effects increases with increasing task demand. They also show that even rather small increases in task demand (here by introducing time pressure) in easy search tasks result in stronger influences of the reward association on performance. The reward-related differences in the intercepts indicate that the reward association increased non-search processing speed in particular. Further, the overall analysis (see Figure 6) revealed that the RT-difference between no and low reward tended to decrease across set-size without time pressure, and the difference between no and high reward tended to decrease across set-size with time pressure. As can be seen in Figure 6, the former effect might be due to a speed-accuracy trade-off for the no reward targets in the set-size 2 condition. Such trade-offs are usually related to changes in the response decision criterion (e.g., Ratcliff and Rouder, 1998). Since participants could not win anything in this condition, it is possible that they strategically responded fast if they saw the no reward color at the expense of an increasing error rate. In the condition with time pressure, a small speed accuracy trade-off can also be seen for high reward targets in the set-size 8 condition. Here, participants made less errors at the expense of an increase in RT. This might be taken as an indication for a delay in responding due to response caution (see Anderson, 2015a, for a similar idea). Such a criterion adaptation makes sense if considering the increase in noise in the set-size 8 condition due to the greater amount of distractors and the higher reward that is at stake.

Importantly, by reducing task demands, contrary to Experiment 1, the relative reward effect remained constant across RTs. The results observed across both experiments also indicate that reward-related effects on performance increase with increasing demands on the search process. This supports the search disadvantage assumption which states that, if task demands increase, more effort is necessary to find the low- or no-reward targets. At the same time, the pattern is contrary to the one that would have been predicted, if the reward-related colors are additionally prioritized top-down as has been assumed within the top-down enhancement assumption.

## General Discussion

In this study, we investigated (a) whether Sha and Jiang’s results (Sha and Jiang, 2016, Experiment 2) are conceptually replicable and (b) if so, how this can be explained. In Experiment 1 (demanding search task), and in Experiment 2 (pop-out search task), participants had to look for one out of three reward-associated colors to find the target. Like Sha and Jiang (2016), we varied line orientation in the distractors and the targets within the same orientation set (horizontal/vertical). In Experiment 1, we observed reliable reward-related performance benefits in the conditions with and without time pressure. In Experiment 2, these benefits only emerged with time pressure. Interestingly, the benefits emerged primarily for high-reward targets, and much less so for low-reward targets. The latter elicited reward-related benefits only with time pressure in Experiment 1. In the following, we will discuss possible underlying mechanisms and processes.

### Search Efficiency and Non-search Processing Speed

In Experiment 1, the flattening of the search slopes with increasing reward shows that search efficiency was boosted by reward. Additionally, a reward-related difference in the intercepts was observed, indicating that non-search processing speed was generally increased by reward. In Experiment 2, where we used a pop-out search task, search slopes were flat, suggesting optimal search efficiency (cf. Lee and Shomstein, 2014; Wolfe and Horowitz, 2017). However, in the condition with time pressure, reward-related search benefits were still present across set-sizes, suggesting reward-dependent increases in non-search processing speed.

These results have three important implications. First, increases in search efficiency can be due to a more effective attentional guidance to the target and to a reduced time necessary for processing the selected stimuli (cf., Wolfe, 2016; Wolfe and Horowitz, 2017). Wolfe and Horowitz (2017) suggested that attention is guided by the color of the reward-associated stimuli, and that the reward association can modulate this guidance. One possibility how this modulation might take place is that features associated with reward become more salient over time (e.g., Hickey et al., 2010). Adopting this perspective, the reward-dependent increase in search efficiency in Experiment 1 suggests that the colors associated with (especially high) reward become more salient. Our results also indicate that this increase in salience might even depend on the amount of associated reward. Since it was also shown that differences in salience can further influence performance if search efficiency is already near the optimum (e.g., Zehetleitner and Müller, 2010), the reward-dependent differences in the intercepts of Experiment 1 and 2 also support this assumption.

Second, in Experiment 2, reward-related search benefits were only found in the condition with time pressure. Time pressure presumably increases the task demands, resulting in more necessary effort to solve the task (cf. Kahneman, 1973). Thus, this result supports the assumption that reward-related performance effects increase with increasing task demand.

Third, reward-dependent increases in non-search processing speed were larger under time pressure than without time pressure, especially for the low-reward condition in Experiment 1 (see Figure 2). Interestingly, this result can be seen as support for the top-down enhancement assumption since one possible explanation is to assume that time pressure generally increases attentional focusing or effort. Kahneman (1973) discussed the close relationship between time pressure, attentional processing, and effort in detail, and there has since been a lot of evidence supporting the notion that time pressure evokes increased attentional effort in a variety of tasks (e.g., Maule et al., 2000; Raymond and O’Brien, 2009; Hübner and Schlösser, 2010). From this perspective, time pressure increases the reward effects by recruiting additional attentional resources, which then boost search performance accordingly. Regarding the question of which mechanisms are at the core of such effort-related changes in attentional processing, an interesting suggestion comes from a study by Milosavljevic et al. (Milosavljevic et al., 2010; see Manohar et al., 2015, for a similar idea). Based on a sequential sampling model, they proposed that reward-related changes in processing under time pressure might result from a noise reduction in the drift process, which may be interpreted as an increased attentional focusing due to stronger attentional capture.

### Modulation of the Relative Reward Effect by Search Time

In both experiments, we investigated how the relative reward effect changed as RT increased. Investigating the relative reward effect has two important advantages. First, it excludes scaling effects (i.e., reward effects increase simply due to increasing RTs). Second, as Watson et al. (2019) pointed out – although for test phases – the difference between high and low reward can just be traced back to their reward history. Other comparisons might be confounded with other aspects of selection history.

In Experiment 1 (demanding search task), the relative reward effect increased over the first four vincentiles in the set-size 2 condition and the first three vincentiles in the set-size 8 condition. Additionally, the effect also increased with increasing set-size. In Experiment 2 (pop-out search task), though, the relative reward effect did not differ across vincentiles irrespective of time pressure and set size. This result pattern across experiments indicates that the relative reward effect increased with increasing task demands and corresponding search duration. This can be explained by the search disadvantage assumption: If the target color is associated with high reward, attention is efficiently directed to this target. This search benefit is rather small in easy search tasks. In more demanding tasks, however, the influence of the reward association on performance increases, since attention is still relatively efficiently guided to the high-reward target, but more effort is necessary to find the low- or no-reward targets relative to the easier search task.

As mentioned above, we also found evidence that the reward-associated colors might be additionally prioritized top-down. This raises the question why we did not find support for the top-down enhancement assumption in the distribution of the relative reward effect in Experiment 2? One possibility is that the influence of top-down enhancement is rather small and can only be observed in very demanding tasks. This is supported by the result that reward-related changes in non-search processing speed were larger under time pressure relative to the condition without time pressure, especially for the low-reward target in Experiment 1 (see above). Alternatively, the reward-related colors were not prioritized top-down since in Experiment 2 a different *search mode* (Bacon and Egeth, 1994; see Gaspelin and Luck, 2018, for a review) could be used to solve the task. In Experiment 1, participants could adopt the so-called *feature search mode*, i.e., they could use an attentional set for the feature characterizing the target, since neither the target color nor the orientation bar were singletons (i.e., stand out in one feature). In Experiment 2, the color popped-out of the search array. Thus, participants could adopt a *singleton detection mode*, i.e., they simply search for a deviating target to solve the task. In sum, participants need an attentional set for the reward-related colors in Experiment 1, while they do not in Experiment 2. As a consequence, participants might not have adopted an attentional set for the different reward-associated colors in Experiment 2, resulting in the observed lack of top-down enhancement. While we cannot exclude this possibility, we consider it unlikely for the following reason: while the specific reward-related colors are not important for solving the task, they are nevertheless important for the main goal of the participants, i.e., earning as much money as possible. Thus, from this more global perspective, the color-reward associations still play a crucial role in solving the task. This thought, however, is not a new one. Bacon and Egeth (1994) also considered that motivational aspects might influence participants in adopting the feature search mode. It is important to note, however, that the question of how specific motivational and selection history aspects influence adopting specific attentional sets is beyond the scope of this study. It is thus for future research to investigate this issue.

### The Role of Line Orientation

In the end, the question arises why in most training-test studies no reward-related performance benefits were observed in training (e.g., Roper et al., 2014; Anderson, 2015a,b), while Sha and Jiang (2016) and we found such benefits? Like Sha and Jiang (2016), we varied the target and distractor lines on the same orientation set (horizontal/vertical), while in most studies, different orientation sets were used for distractor and target lines, respectively. In the latter variant, the search task is less effortful, since the targets stand out due to their color *and* their line orientation. Thus, task demands might be a relevant factor influencing reward-related performance effects. This is supported by two results. First, the relative reward effect increased with search duration in a demanding search task, but not in a less demanding pop-out search task. Second, in Experiment 2 (pop-out search task), reward-related performance benefits were only observed if additional time pressure was introduced.

Although the increased task demands might explain the reward effects in the training of Sha and Jiang (2016), the results of Experiment 2 indicate that this might not be the only reason, why in many studies no effects emerged: In a less demanding pop-out search task, we observed significant reward-related benefits in the offset of the search function with time pressure (see also Kiss et al., 2009; but see Wang et al., 2014, for contrary results), and a tendency toward such effects without time pressure (see also Lee and Shomstein, 2014, who observed reliable effects in this condition even without time pressure).

Varying the lines within the target and the distractors on different orientation sets not only decreases the task demands, it might result in participants using a different search strategy: In our study, participants could only solve the task if they searched for the reward-related color. In most studies, though, participants could also find the target by searching the uniquely oriented line. Although vertical/horizontal lines are not supposed to pop-out of tilted lines (cf. Treisman, 1985; Theeuwes, 1991), Lee and Shomstein (2014) nevertheless observed that horizontal/vertical lines are found rather efficiently among tilted ones. Finding the unique line might be easier than searching for the reward-related color among other colors. Thus, the absence of reward-related effects in the training sessions of most of the previous studies might also be due to participants using the nonrewarded orientation feature to solve the task. However, here we focused on the conceptual replicability of the results of Sha and Jiang (2016) in their training and how task demands might modulate reward effects. Thus, it is to future research to investigate the role of line orientation and the associated strategy use.

### Summary

Similar to Sha and Jiang (2016, Experiment 2), we observed reward-related performance benefits in a (training) search task. By using a demanding search task, we found that the reward association of a stimulus influenced its non-search processing speed and the efficiency of the search process. In a pop-out search task, if search efficiency was already optimal, non-search processing speed was still influenced by reward association, however, only so if time pressure was introduced. Since more effort is needed to solve a task under time pressure (cf. Kahneman, 1973), this result indicates that the influence of reward-related stimuli on performance increases with increasing task demands. Our results also show that the reward-related performance effect increased with increasing search duration in demanding tasks. But if the target popped-out of the search array due to its color alone, search duration had no influence on the reward effect. This indicates that in demanding tasks, the reward-related benefit remains stable with increasing search duration, but more effort is necessary to find stimuli associated with less or no reward, resulting in a search disadvantage. Moreover, we found evidence that the reward-related color might be additionally prioritized top-down in very demanding tasks. Thus, our results suggest that reward-related attentional biases might be especially powerful with increasing task demands.

## Data Availability Statement

The raw data supporting the conclusions of this article will be made available by the authors, without undue reservation.

## Ethics Statement

Ethical review and approval was not required for the study on human participants in accordance with the local legislation and institutional requirements. The patients/participants provided their written informed consent to participate in this study.

## Author Contributions

AW and MD: literature review, idea generation, formulating hypothesis, design of behavioral experiments, data analysis, interpretation of results, preparation of draft manuscript, and preparation of final manuscript. All authors contributed equally to the article and approved the submitted version.

### Conflict of Interest

The authors declare that the research was conducted in the absence of any commercial or financial relationships that could be construed as a potential conflict of interest.

## Footnotes

^1^A detailed description about the relationship between task demands, the increase in attentional effort also with regard to the presence of motivational incentives as well as the corresponding recruitment of top-down control processes can be found in Sarter et al. (2006).
